# Symposium on Lactic Acid Bacteria—reading while waiting for a meeting

**DOI:** 10.1093/femsre/fuaa049

**Published:** 2021-03-16

**Authors:** Bas Teusink, Oscar P Kuipers, Sylvain Moineau

**Affiliations:** Amsterdam Institute of Molecular and Life Sciences (AIMMS), Vrije Universiteit Amsterdam, O2 building, location code 2E51, De Boelelaan 1085, NL-1081HV Amsterdam, The Netherlands; Molecular Genetics, University of Groningen, Nijenborgh 7 (Linnaeusborg, building 5172, room 6.50), 9747AG Groningen the Netherlands, The Netherlands; Département de biochimie, de microbiologie, et de bio-informatique, Faculté des sciences et de génie1045, avenue de la MédecineUniversité Laval, Québec, Canada, G1V 0A6

This special thematic issue of *FEMS Microbiology Reviews* is truly special, because it contains contributions to a meeting that is yet to happen! As many others, the thirteenth International Symposium on Lactic Acid Bacteria (LAB13) was a victim of the COVID-19 pandemic and has been postponed to next year. The conference is held every 3 years in The Netherlands, and is attended by researchers from academia and industry from all over the world, reflecting the importance of these microorganisms in food, health and basic science. As a tradition, the invited speakers are asked to contribute not only by a talk, but also by a thorough review on the topic of their presentation. These papers were already under review by the time it became clear that the coronavirus would not be contained and that we had to postpone the meeting. However, we decided to move on and publish the reviews now, when still timely, and we are eagerly awaiting updated presentations next summer.

Lactic acid bacteria, or LAB for friends, are a group of phylogenetically diverse, but metabolically rather similar, bacteria. They play important roles in food and feed fermentations, where they contribute to safe, healthy and tasty food. Some strains have probiotic properties and they are even potential delivery vehicles for (oral) vaccines. They are selected from spontaneously fermented products or through evolution experiments in the laboratory. They are screened for desired flavor-producing properties, or for antimicrobials that they produce to defend their often nutrient-rich niches. They need to defend against (bacterial) viruses, which are a significant risk for industrial food fermentations. They are studied for their metabolism and physiology, in isolation or increasingly in the context of microbial consortia such as the human microbiome or complex fermented foods. These broad topics are discussed at the LAB Symposium series, and this issue reflects the current state of the art of the exciting and diverse field of LAB research.

Research into viruses—bacteriophages in the case of bacterial viruses—has never been more pressing than in current pandemic times, but has been a focal area in LAB research for decades. It is not only a threat to industrial production processes, but fundamental studies on the clustered regularly interspaced short palindromic repeats (CRISPR)-CRISPR-associated (Cas) systems in *Streptococcus thermophilus* led to the discovery of the molecular mechanisms of this adaptive immune system. We have come a long way since then. Roberts and Barrangou discuss the history, the different systems and their mode of action and highlights the applications of the CRISPR-Cas system in the context of LAB.

While CRISPR-Cas systems may limit horizontal gene exchange in some LAB species, bacteria have still numerous ways to exchange genes as mobile genetic elements are drivers of microbial evolution. Various mechanisms of horizontal gene transfer can also occur in microbial communities. However, monitoring DNA transfers and their effects in these communities has been challenging. Christina Saak and colleagues explore existing and emerging technologies for tracking mobile elements and assigning them to microbes in order to generate comprehensive community-level information. They also make the case that LAB-driven fermented foods are, among others, ideal models to apply these techniques and address outstanding questions in the field of horizontal gene transfer in microbial communities.

Dennis Romero and colleagues address phage–host interactions in *Lactococcus lactis* and *Streptococcus thermophilus*. They provide a timely overview of the diversity of these phages and their biology, as well as the defence mechanisms operating in these LAB. This knowledge has led to improved industrial starter cultures, e.g. specifically designed and adapted to prevent fermentation delays and low-quality fermented products. The authors also describe the oldest and still highly dynamic arms race between prey and predator and its relevance for developing robust dairy fermentation processes.

While phages are arguably the master regulators of LAB communities, they also likely play a role in other ecosystems, including the human gut. The function and composition of our gut microbiome are clearly linked to human health. Consequently, the promise of being able to precisely modulate this microbial community has become an area of intense research worldwide. Torben Rasmussen and colleagues discuss the promises and limitations of fecal microbiota transplantation, which offers an attractive therapeutic strategy for patients suffering from gut dysbiosis and bacterial infections. Growing evidence also suggests that cascading effects are initiated when phage communities are also transferred to the gut, which leads to changes in its microbial composition and the host metabolome as well as improving health.

As the role of microorganisms in intestinal health has become clearer with every LAB symposium due to advanced tools and new databases, we have invited a number of contributions related to this topic. Shaopu Wang and colleagues review the impact of early-life exposures on the microbiota development during childhood, and how this affects health at a later age. They identify ‘golden’ perinatal factors and risk factors that impact on physiology, immunity and even cognitive development. They also describe possible interventions and their potential.

One of the first colonizers of the human gut are bifidobacteria, which are strictly speaking not LAB, but whose LAB-assisted applications, metabolism and impact on gut health warrant a place in this thematic issue. Chyn Wong and colleagues describe the biology and physiology of the Human Residential Bifidobacteria, and what factors contribute to their ecological fitness. These factors may be used for the selection of ‘bifido’ strains with desired properties and health benefits. Strain selection is also essential for the production of healthy fermented foods. Francesca De Filippis and colleagues discuss the role of fermented foods as a source of potentially probiotic LAB, and how to mine the wealth of genomics and metagenomics data with bioinformatics and data analysis tools. While more research is still needed to better understand the health benefits of fermented foods and their associated LAB, the field keeps progressing between each LAB Symposium.

De Vuyst and Leroy looked at fermentation processes and describe in particular how yeasts, acetic acid bacteria and LAB collaborate during the fermentation of cocoa beans, a necessary step to obtain the chocolate flavors we love so much. The complexity of food fermentations is in full display, yet it also shows the great progress made in our understanding of the interactions and the physiology of these microorganisms through a combination of (functional) genomics, controlled fermentations and modeling. Michiel Kleerebezem and colleagues review the latest insights into the physiology of *Lactococcus lactis*, one of the key species in starter cultures for a.o. cheesemaking, and an important model for LAB research. They describe both the functional genomics and bacterial physiology approaches to understand the adaptation of *L. lactis* to different environmental conditions, including zero growth conditions that are relevant for cheese ripening. *L. lactis* was also the first LAB for which a genome-scale metabolic model was developed. Frank Bruggeman and colleagues take us on a journey into the theory of bacterial physiology and growth that forms the rationale for all constraint-based modeling approaches. They show from first principles how the strategy of growth rate (fitness) maximization under biochemical constraints can explain a surprisingly large part of microbial behavior, from *L. lactis* to *Escherichia coli* to yeast. This includes stress resistance, overflow metabolism and catabolite repression.

Finally, as LAB take up many nutrients from the environment, one of the key constraints is the cell wall and membrane. The cell wall is also important for stress-resistant properties and adherence to other cells and surfaces. Martinez and colleagues describe the biochemistry of cell walls in LAB, and discuss the challenges, insults and defenses that LAB have evolved to cope with in the nutrient-rich environments as well as in stressful ones. They show the crucial interplay between cell wall biosynthesis and global cellular metabolism, guiding the plasticity of the wall. Moreover, the important role of the bacterial cell wall in phage adsorption and bacteriocin activity is discussed, showing both a receptor and a defensive role of the cell wall. Overall, this underscores the importance of understanding cell wall structure and function to develop novel applications of LAB in health and biotechnology.

We hope that this thematic issue will be of interest to many researchers inside and outside of the LAB field, and that it will inspire many to attend the thirteenth International Symposium on Lactic Acid bacteria in 2021.
